# Experience of violence, coping and support for nurses working in acute psychiatric wards

**DOI:** 10.4102/sajpsychiatry.v28i0.1700

**Published:** 2022-05-30

**Authors:** Ntombiyakhe Bekelepi, Penelope Martin

**Affiliations:** 1 School of Nursing, Faculty of Community and Health Sciences, University of the Western Cape, Cape Town, South Africa

**Keywords:** acute ward, coping, experience, nurse, support, violence

## Abstract

**Background:**

Acute psychiatric wards are stressful working environments because of the nature of the mental illness of patients admitted. These patients present with a variety of complex psychiatric problems and social control that require skilled and competent nurses to manage them. The shortage of nurses, especially with advanced psychiatric qualifications or necessary experience, may create challenges for nurses as they navigate this stressful working environment.

**Aim:**

The aim of this study was to explore and describe nurses’ experience of patient violence, coping strategies and received support whilst working in acute wards in psychiatric hospitals.

**Setting:**

This study was conducted in six acute wards of the three psychiatric hospitals in Cape Town, South Africa.

**Methods:**

A qualitative, explorative, descriptive design was conducted using semi-structured interviews to obtain data from 14 nurses working in acute wards in three psychiatric hospitals in the Western Cape.

**Results:**

Five themes were generated using thematic analysis: violence perceived to be ‘part of the job’, contributing factors to patient violence, physical and psychological effects on nurses, adaptive and maladaptive coping strategies and perceived support from stakeholders.

**Conclusion:**

Participants normalised patient violent behaviour as being part of the job to minimise the physical and psychological effects of the traumatic experience. Adaptive and maladaptive coping strategies were used to cope with the traumatic experiences of being assaulted by patients. Recommendations allude to practising self-care and attendance of training in the management of aggressive patients for nurses, to enhance a variety of adaptive coping strategies.

## Introduction

The incidence of violence towards healthcare staff in psychiatric wards, including nurses, is repeatedly depicted as high in comparison with other health care environments.^1^ However, many healthcare staff members consider patient violence to be a routine part of their work, thus normalising violence.^2^ Violence is defined as the:

[*I*]ntentional use of physical force or power, threatened or actual, against oneself, another person or against a group or community which either results in or has a high likelihood of resulting in injury, death, psychological harm.^3^

Nurses, as front-line workers working in acute psychiatric wards, are in close contact with patients and their families, resulting in numerous interactions that make them at risk of encountering violent incidents.^2,4^ Patient physical assault of staff members is disturbingly prevalent in psychiatric settings,^5^ with 36.4%^6^ and 95%^7^ of nurses experiencing physical assault. Lozzino et al.^8^ purported that one in five patients admitted to acute psychiatric wards may commit an act of violence, which may impact the quality of care.

Being exposed to routine violence can overwhelm the usual coping strategies used by nurses and reduce cognitive, emotional and behavioural performance that might negatively affect the quality of care.^9^ The present study aimed to explore and describe nurses’ experiences of patient violence in acute psychiatric wards, coping strategies and perceived support following violent incidents.

## Research methodology

### Study design

A qualitative, exploratory and descriptive design was used in this study.

### Setting

This study was conducted at six acute wards of three psychiatric hospitals in Western Cape. The wards are 30-bedded wards. Most of the patients were admitted involuntarily as they present a danger to themselves and others.^10^

### Study population and sampling strategy

The study population included all nursing categories (professional nurses, enrolled nurses and enrolled nursing assistants) working at the participating hospitals. A purposive sampling was used to select 14 nurses from the nursing categories working in acute wards providing direct patient care and who had experienced physical violence from patients.

### Data collection

Data were collected from August to October 2019. Twelve females and two males participated in the study. A semi-structured interview guide was used with the following questions. (1) Can you describe your experience of being assaulted by a patient? (2) Describe how you felt after the incident. (3) What help did you receive after the incident of assault? Probes were used to elicit clarity on responses. The interview guide was piloted with two participants, who were working in an acute ward in one of the selected psychiatric hospitals, to check if the questions were clear and whether they elicited information to meet the aim of the study. The interviews were conducted in a private room in the research settings. All interviews were audiotaped with the permission of the participants.

Trustworthiness was enhanced by the use of Lincoln and Guba’s framework as cited in Creswell,^11^ which included criteria of credibility, transferability, dependability and confirmability. Member checking was done by sending transcripts to participants for validation if there was information that they wanted to add or remove if it did not reflect their experience. The research supervisor acted as an independent coder. The researcher bracketed preconceived ideas about violence by psychiatric patients to ensure that the findings only represented the views of the participants. To ensure the confirmability of results, excerpts from the transcribed interviews were used in the description of the findings.

### Data analysis

All audio-recorded interviews were transcribed verbatim. Transcribed data were imported into Atlas.ti 8 software. Thematic analysis following the criteria by Braun^12^ was used to analyse the data. Five themes were generated depicting nurses’ experience of patient violence, coping and perceived support whilst working in acute wards at three psychiatric hospitals.

### Ethical considerations

Ethics approval was obtained from the BioMedical Research Ethics Committee in one of the public universities in Cape Town, Western Cape (Reference No.: BM18/6/18) and the Western Cape Department of Health (Reference No.: WC_201809_005). Permission to conduct the study was obtained from the head of each hospital. Informed consent was obtained from all the participants, and participants were informed that participation in the study was voluntary. Participant information sheets were disseminated to all participants explaining the aim, ethical considerations and guidelines for participation in the study. The researcher ensured that the names of participants were not linked to the data to ensure anonymity. Transcriptions were kept locked in a safe place to maintain confidentiality.

## Results

Participants’ demographic information is provided in Table 1. Most of the participants were female. The participants’ registration category as a nurse with the South African Nursing Council varied.

**TABLE 1:** Participants’ demographic data.

Participant	Gender	Age (years)	Years of experience	Nursing category
LP1	Female	28	5	Professional nurse
LP2	Female	44	18	Professional nurse
LP3	Female	58	33	Professional nurse
LP4	Female	28	4	Enrolled nursing assistant
LP5	Female	57	33	Enrolled nurse
SP6	Female	37	7	Enrolled nursing assistant
SP7	Female	31	4	Enrolled nursing assistant
SP8	Female	56	30	Professional nurse
VP9	Male	34	5	Enrolled nursing assistant
VP10	Female	43	5	Professional nurse
VP11	Female	56	29	Professional nurse
VP12	Male	39	9	Enrolled nursing assistant
SP13	Female	35	4	Enrolled nursing assistant
SP14	Female	35	10	Professional nurse

## Themes and categories

Five themes and 16 categories emerged from the analysis of the data and are depicted in Table 2.

**TABLE 2:** Participant themes and categories.

Themes	Categories
1. Violence perceived to be part of the job	Justified patient violent behaviour as patients were perceived to be right. Being violent was seen as part of the illness. Dealing with patients’ violent behaviour was seen as an expectation of the job. Hesitant in reporting incidents because of the way they were dealt with.
2. Contributing factors to patient violent behaviour	Inability to access nicotine because of smoking policy changes. Diagnosis, history and the type of patient
3. Physical and psychological effects on nurses	Physical injuries and psychosomatic effects of the trauma were reported by participants. Physiological and psychological signs and symptoms in response to violence by patients.
4. Adaptive and maladaptive coping strategies	Sharing ways of dealing with traumatic experiences at work and finding help to cope with the situation. Using humour to cope with violent incidents. Hypervigilant around patients. Minimising the effects of violence and withdrawal from patients.
e. Perceived support from stakeholders	Formal support received from supervisors. Support from family and colleagues. Perceived lack of support from management, which when given was perceived to be unsatisfactory. Interrogation following an incident. Training on management of aggressive patients after incidents.

### Theme 1: Violence perceived to be part of the job

Participants appraised the environment by justifying the violent behaviour displayed towards them by patients as an inherent attribute of working in acute psychiatric wards. The nature of the work raised an expectation amongst the participants that they may be exposed to violent behaviour or incidents displayed by patients who could not be held accountable for their behaviour. This has been noted by the participants who said:

‘But I mean the patient is psychotic … it is part of her illness.’ (LP3, female, 58 years, professional nurse)

‘You must know how to treat your patients and the patient is right all the time.’ (LP3, female, 58 years, professional nurse)

Violent incidents were experienced as challenges as participants normalised the patient’s behaviour. Regardless of the working environment, participants did not feel the need to be moved to another ward.

‘Because it’s part of your job … You must just carry on with your duties.’ (VP11, female, 56 years, professional nurse)

‘… [*F*]or me it’s like I know I am working in this kind of environment, so it can happen at any time.’ (SP6, female, 37 years, enrolled nursing assistant)

‘No, I didn’t even think of going out of the unit, yeah, I was just holding on and waiting for the next challenge to come.’ (VP10, female, 43 years, professional nurse)

Violence was an expectation that was deemed to be part of the unsafe working environment.

‘I don’t feel safe as I did …. in psychiatry you can never feel safe, but now I’m more cautious.’ (LP1, female, 28 years, professional nurse)

### Theme 2: Contributing factors to patient violent behaviour

The changes in hospital smoking policy, patient diagnosis and type of admission were identified as factors contributing to patient violent behaviour. Participants reported that when patients were unable to access cigarettes, they became violent. Participants felt the restrictions that had been posed by the implementation of the new rules on smoking times had contributed to patient violent behaviour, as noted by the following participants:

‘I remember on that particular night he wanted a smoke, and we have rules in the unit … and it wasn’t a smoking time.’ (LP2, female, 44 years, professional nurse)

‘First of all, our patients are addicted to tobacco … if I can say … let me just say psych patients are addicted to tobacco … So … they can’t cope without smoking.’ (VP9, male, 34 years, enrolled nursing assistant)

Participants identified various mental illnesses and associated symptoms as contributing to patient violent behaviour. Accompanying the mental illness were patients who had a history of violent behaviour towards staff members.

‘Yes, definitely, there is a difference; most of our patients come now with substance-induced psychosis and display challenging behaviour.’ (VP10, female, 43 years, professional nurse)

‘Oh … is mostly the aggression … especially with bipolar mood patients when they do not like you, they don’t like you, that was one of them and the other thing is when they are delusional.’ (LP3, female, 58 years, professional nurse)

‘We had this one patient, who is one of our chronic [*patients*], that has a tendency of choking people whether [*it*] is patient or staff member, that was the patient’s habit.’ (LP2, female, 44 years, professional nurse)

Involuntary admission of patients who were a danger to themselves and others was identified as contributing to violence towards the participants.

‘The patient was aggressive and because she was an involuntary admission, she was not keen to come in.’ (LP4, female, 28 years, enrolled nursing assistant)

### Theme 3: Physical and psychological effects on nurses

Post-exposure to experiencing patient violence manifested in physical and psychological effects on nurses.

Participants reported having suffered injuries, such as bruises, bites and punches during physical attacks by the patients. These attacks were unexpected as they occurred suddenly without provocation. Some of the attacks left participants permanently physically disabled.

‘She grabbed my finger and she bit me on my thumb, and she didn’t let go. I could feel the teeth in my flesh, and we couldn’t let go.’ (SP8, female, 56 years, professional nurse)

‘I never thought in my mind that I would ever be assaulted by a patient, and I’m permanently disabled. I’m limping and I’m not well at all.’ (VP12, male, 39 years, enrolled nursing assistant)

‘As I was talking to her, she did not answer, instead she punched me in the stomach.’ (SP7, female, 37 years, enrolled nursing assistant)

Participants described the trauma experienced during a violent incident where they were attacked by patients. Participants reported trauma-related thoughts of pending death and accompanying anxiety following the violent incident.

‘Yhoo! I actually thought I’m gonna die, I’m not lying, like I said we were, I was … I was standing up straight but from behind he got and … as he held me, we were going down on the floor (participants demonstrated).’ (LP1, female, 28 years, professional nurse)

‘I think I was feeling so hot and bothered, sweating, anxious, hmm … overwhelmed, still in disbelief.’ (LP2, female, 44 years, professional nurse)

‘I was a bit nervous, but I couldn’t allow him to see that I was nervous, he came to me to apologise after the incident, but the first 3 days I felt that … you know that … that jittering feeling you get in your stomach.’ (LP2, female, 44 years, professional nurse)

Participants described the fear they experienced when they were attacked by patients. The inability to access help further exacerbated the feelings of hopelessness as the people who were meant to protect them, such as the security officers, were deemed unable to do so. An intense emotional state of anger and of being violated resulted in a participant being absent from work for a lengthy period of time to deal with the effects of the incident.

‘Oh yeah … I felt hopeless, that was one of the things, hopeless because I couldn’t really protect myself or the security couldn’t help me.’ (LP3, female, 58 years, professional nurse)

‘I was scared because maybe … I don’t know what is on the patient’s mind, I don’t know what can happen again, or what.’ (SP6, female, 37 years, enrolled nursing assistant)

‘Yoh! I was angry, I was really angry, I felt so violated and I felt so … [*sighs*] I’m not coming to work for a whole month.’ (SP8, female, 56 years, professional nurse)

### Theme 4: Adaptive and maladaptive coping strategies

The study participants shared their ways of coping with the traumatic experience. These included talking to colleagues about their experiences and humour as adaptive coping strategies, minimising the seriousness of the incidents and the effects and withdrawal from patients as a maladaptive coping strategy. They further alluded to time being a healer of traumatic experiences.

‘I did cope well because I did speak to my colleague about it.’ (LP5, female, 57 years, enrolled nurse)

‘Yeah … that’s how you relieve your stress …. we also joke about it afterwards.’ (LP3, female, 44 years, professional nurse)

‘What is also helping me is that I talk about it a lot even with my colleagues … and even talking to strangers like you do helps in a way.’ (VP11, female, 56 years, professional nurse)

‘It wasn’t as bad as I thought it would be afterwards … I wasn’t very traumatised, I would say, but we were just taking safety precautions, yeah.’ (LP1, female, 28 years, professional nurse)

Participants reported that they tended to be hypervigilant when around patients and distanced themselves from some known patients with violent behaviour. They also reported that they became cautious when patients became overfamiliar, so withdrawal was used as a form of coping in situations like these.

‘It made me, ah … keep a distance from them, yes, because sometimes the patients just want to come to you and maybe grab you.’ (LP5, female, 57 years, enrolled nurse)

### Theme 5: Perceived support from stakeholders

Some participants reported a lack of support following an incident, whilst others reported that they were not satisfied with the support provided, as top management seemed not to care about their well-being. Some participants reported that they have received support from managers, family, friends and colleagues as they felt these people were always available to talk to.

‘My operational manager was quite supportive … She was also angry that we have worked so hard, and we care for those people, and this is what happened and that they hurt you for no reason, but she gave me a lot of support really.’ (SP8, female, 56 years, professional nurse)

‘But my children were very supportive then and even now.’ (VP11, female, 56 years, professional nurse)

‘I got all the support I needed, right, but what hurt me, top level from the area manager, I didn’t … you know, I feel that is very important as a person working in a hospital, just that little phone call … you know, and that hurt because that I didn’t get, that was the only thing.’ (LP1, female, 28 years, professional nurse)

The apparent, perceived lack of management support made participants feel that they were the cause of patient attacks on them. Incidents were not reported to management, as participants reported being interrogated about their contributing role to the violent behaviour by patients.

‘The action they take when they investigate the incidents, they always ask staff “what have you done to the patient?” Although they know the patients that we are dealing with, and people are reluctant to report incidents because they know they will be cross-questioned.’ (VP9, male, 39 years, enrolled nursing assistant)

‘I would be lying if I’m saying the manager ever phones to check up on me when I told them I have depression.’ (SP13, female, 35 years, professional nurse)

Prior to working in an acute psychiatric ward, training on the management of violent patients was identified as a need. One participant reported being sent for training on management of violent patient behaviour after an incident.

‘I was only sent for training after the incident … maybe it was done for the future as well, as that could happen again.’ (LP4, female, 28 years, enrolled nursing assistant)

Accessibility to the staff support programme, Independent Counselling and Advisory Services (ICAS), was limited when participants experienced violent incidents. They reported requiring immediate intervention, which was perceived to be unavailable. Some participants organised their own counselling. The need for an in-house, hospital-based psychologist was identified to provide immediate support to staff members who had experienced violence.

‘My experience with ICAS is when you phone them, you want to see them tomorrow or they make an appointment. I want somebody to be there now… then they come to see you, by that time I’m away.’ (SP8, female, 56 years, professional nurse)

‘I did organise the counselling myself outside of the facility, but the facility itself did not.’ (VP10, female, 43 years, professional nurse)

‘I think it was going to be better if maybe at the hospital we have psychologists that are there for the staff.’ (VP10, female, 43 years, professional nurse)

## Discussion

Nurses in this study detailed their traumatic experience following exposure to patient’s violence in the acute ward, alluding to these incidents as expected when working in acute units. Participants identified perceived factors contributing to incidents, the effects of these incidents and various individual and organisational support strategies.

Nurses working in psychiatric settings are frequent victims of workplace violence, most of which are perpetrated by patients.^13^ In the current study, participants perceived the culture of the psychiatric ward as one where patient violence was accepted as part of the job and unavoidable, often justifying this behaviour of patients as part of their mental illness. This may hinder the reporting of these incidents, as they deemed them not important. Moylan et al.^14^ stated that when staff members see violence as part of the job, they do not recognise the need for reporting, which may have an impact on the provision of support. Zuzelo et al.^15^ alluded that being exposed repeatedly to violence may increase the likelihood of desensitisation. Despite being assaulted by patients, the participants reported that they were expected to continue with work. Similarly, participants in Yang et al.’s study^16^ reported that their expected roles as nurses had to continue caring and maintaining contact with patients who assaulted them.

Participants identified various contributing factors to patient violence in the acute wards. These included changes in smoking policy that had an impact on the accessibility of nicotine in hospital settings. Similar findings were reported in a study on a trial of smoke-free policy in an acute mental health unit where participants reported an increase in physical injuries because of patient violence.^17^ Although not all patients or even patients with a history of violent behaviour may display violent behaviour,^16^ the participants reported that in their experience, patient diagnosis and associated symptoms were contributing factors to patient violence. These findings are consistent with that of Nguluwe et al.,^18^ where participants reported mental illness as one of the reasons for patient violence. Most participants in the study reported that these violent incidents by patients happened suddenly without any provocation, and patients were seen as unpredictable. Similarly, Yang et al.^16^ reported that patients are unpredictable, and these assaults occur suddenly during ordinary contact.

Looking at the forms of physical violence experienced, the findings alluded that participants were punched, choked, kicked or slapped. Similar findings were reported by Nguluwe et al.,^18^ where participants reported being beaten, slapped, bitten, grabbed and pushed. In this study, participants suffered physical injuries such as fractured bones, bruises, torn tissues and permanent physical impairment. Consistent with this, Stevenson et al.^2^ reported that participants were suffering from negative physical health following an exposure to physical violence, such as bruises, bites, musculoskeletal shoulder and knee injuries, headaches and muscle tensions.

Fear, shock, anger and disbelief following an exposure to violence were reported in this study. Fear was described by the participants as they felt helpless and did not know what the patients were capable of and what could happen if they failed to manage the situation. Similarly, Maluleke et al.’s study^19^ showed that when nurses experience violence, they become fearful of patients and make protecting themselves a priority, which may impact patient care. Anger towards perpetrators of violence was also reported, which made participants feel like staying at home and not facing the patients. Stevenson et al.^2^ also reported anger towards patients following an incident and that it was most prevalent if staff members perceived that the patient could have controlled their behaviour. More than fear, some participants reported having suffered from mental illnesses such as depression and post-traumatic stress disorder (PTSD) after being assaulted by a patient. Similarly, Dean et al.^20^ reported that participants showed signs of depression, anxiety, burnout and PTSD which affected their personal and professional lives.

Coping is defined as ‘constantly changing cognitive and behavioural efforts to manage specific demands that are appraised as exceeding the resources of a person’.^21^ Happell et al.^22^ suggest that if stressors cannot be avoided in the working environment, nurses need to be assisted with developing adaptive coping strategies to improve their lives. Participants in this study used adaptive or maladaptive coping strategies to mitigate the effects of violent trauma they experienced. Peer support by talking about the experience assisted participants to cope with the traumatic experience. Similarly, participants in Niu et al.’s study^4^ reported that talking to family and friends released their emotions after an exposure to violence. Withdrawal as a form of coping was reported by participants to ensure their safety. This type of coping may have a negative effect on the quality of care rendered to patients as staff members avoid interacting with the patients that assaulted them. Similarly, participants in Stevenson et al.’s study^2^ reported that to ensure their safety, they physically distanced themselves from patients when they were assigned to provide nursing care to the perpetrator.^2^

Some participants in the study reported receiving support (phone calls and messages) from their supervisors, family and friends following a violent incident. Others verbalised their disappointment in the lack of support from top management following a violent incident. Consistent with this study, Stevenson et al.^2^ reported that nurses described feeling angry, unsupported and blamed by their managers, whilst others reported that the managers showed support by means of phone calls which were perceived to be thoughtful. Similarly, Yang et al.^16^ reported that participants sought informal support from other staff members as managers at times did not fulfil their need for support.

It became evident in the current study that participants underutilised the existing formal support for which they gave reasons. Some participants said it is the responsibility of their managers to provide the support required when exposed to violence and noted the timing of referrals and the response from the service provider. Although the hospitals paid for staff support services, it was deemed unavailable, and recommended that hospitals have a hospital-based psychologist. They believed that psychologists would have a better understanding of the challenges they encounter. Participants in this study indicated to have organised their own counselling because of a perceived lack of support as they could not cope after the violent experiences. Similarly, Zhao et al.^23^ reported that participants received support from colleagues, family and friends following an exposure to violence but a lack of support from management. Staff support procedures following a violent incident were noted by Cooper et al.^24^ as a concern. The timing of follow-up was hindered by staff members being off duty after the incident. Support may not have been needed upon return to work.

Training of staff members on management of aggressive patients prior to working in the acute ward was deemed necessary. However, training as a preventative measure was underutilised, with only one participant reporting having been sent for training in management of aggressive patients following an assault. Timori et al.^25^ expressed the need for training to manage patients in acute wards to reduce the risk of injuries.

## Conclusion

Nurses working in acute psychiatric wards experienced physical violence from the patients; however, they normalised the behaviour. Adaptive and maladaptive coping strategies were used in coping with traumatic experience. Recommendations allude to the use of self-care and organisational support by means of the training in the management of aggressive patients to enhance adaptive coping strategies.

## Limitations

Because of time constraints and limited resources, this study only included acute wards in three psychiatric hospitals, which limit the generalisability of the findings. Secondly, the sample of the study was mostly female nurses. These findings should be treated with caution as coping strategies might be different for both genders.
